# The implementation research institute: training mental health implementation researchers in the United States

**DOI:** 10.1186/1748-5908-8-105

**Published:** 2013-09-05

**Authors:** Enola K Proctor, John Landsverk, Ana A Baumann, Brian S Mittman, Gregory A Aarons, Ross C Brownson, Charles Glisson, David Chambers

**Affiliations:** 1 Center for Mental Health Services Research, George Warren Brown School of Social Work, Washington University in St. Louis, 1 Brookings Drive Campus, Box 1196, St. Louis, MO 63130, USA; 2 Child and Adolescent Services Research Center, San Diego Children’s Hospital, San Diego, CA, USA; 3 VA Greater Los Angeles Healthcare System, Sepulveda, CA, USA; 4 Department of Psychiatry, University of California, San Diego, CA, USA; 5 Washington University School of Medicine, Washington University in St. Louis, St. Louis, MO, USA; 6 Children’s Mental Health Services Research Center, University of Tennessee, Knoxville, TN, USA; 7 Division of Services and Intervention Research, National Institute of Mental Health, Bethesda, MD, USA

**Keywords:** Dissemination research, Implementation research, Training, Translational research

## Abstract

**Background:**

The Implementation Research Institute (IRI) provides two years of training in mental health implementation science for 10 new fellows each year. The IRI is supported by a National Institute of Mental Health (NIMH) R25 grant and the Department of Veterans Affairs (VA). Fellows attend two annual week-long trainings at Washington University in St. Louis. Training is provided through a rigorous curriculum, local and national mentoring, a ‘learning site visit’ to a federally funded implementation research project, pilot research, and grant writing.

**Methods:**

This paper describes the rationale, components, outcomes to date, and participant experiences with IRI.

**Results:**

IRI outcomes include 31 newly trained implementation researchers, their new grant proposals, contributions to other national dissemination and implementation research training, and publications in implementation science authored by the Core Faculty and fellows. Former fellows have obtained independent research funding in implementation science and are beginning to serve as mentors for more junior investigators.

**Conclusions:**

Based on the number of implementation research grant proposals and papers produced by fellows to date, the IRI is proving successful in preparing new researchers who can inform the process of making evidence-based mental healthcare more available through real-world settings of care and who are advancing the field of implementation science.

## Background

Advancing the science of implementation is a priority in mental health, where disease burden is high, the repertoire of effective treatments is growing, but availability and receipt of evidence- based care is low. A summary of epidemiologic survey data suggests that only 40% to 50% of people with mental disorders receive any treatment and of those receiving treatment, a fraction receive what could be considered ‘quality’ treatment [1-4]. The gap between what could be delivered and what people in need of mental health services receive—a direct focus of implementation science—is vast [5]. While the pace of implementation science development has increased, we still see comparatively little investment in health services research and even less in the more specific area of implementation science [6].

To accelerate the implementation of evidence-based mental healthcare, the field must meet two pressing needs: a body of research and theory informing effective implementation processes, and a research workforce capable of conducting rigorous and relevant implementation studies. Over the past decade, the field has experienced what National Institute of Health’s (NIH) Dr. David Chambers characterized as an ‘explosion’ in quality and quantity of implementation research [7]. This is demonstrated by ‘rigor and ambitiousness of ongoing studies,’ more comparative effectiveness studies of active [dissemination and implementation] strategies,’ and the ‘capacity building of multiple research centers and networks’ [7]. This paper focuses on the necessary complementary efforts to grow the implementation science workforce. It describes the rationale, components, outcomes to date, and participant experiences with the National Institute of Mental Health (NIMH) funded Implementation Research Institute (IRI) in mental health at Washington University in St. Louis.

### Background, institute aims, and overview

In 2004, the NIH began convening meetings to advance implementation science, but no NIH-funded training programs (*e.g.*, T32 programs, other R25 programs) focused squarely on this field. Recognizing the dearth of investigators prepared to meet the unique conceptual and methodological challenges in implementation science, the authors of this paper began working together in 2006 to address the need for specialized training, consider various training approaches, and explore mechanisms of funding support.

Several principles and guiding assumptions shaped our implementation science training plans. First, because implementation science was still emerging with rapid and continuous knowledge developments for the foreseeable future, its training would need to differ from that in other fields which could immerse trainees in a mature science. We agreed that training would need to be informed by the continuous refreshing and infusion of new intellectual capital. This would require annual reassessment and possible revision of the curriculum, as well as change in roster of training faculty. The Core Faculty accepted a second guiding principle: that our efforts required us not only to ‘stay on top of’ a rapidly developing field, but also to contribute to the field’s advancement. Thus we agreed that the training endeavor itself should help accelerate the development of intellectual capital for the field. Accordingly, the Core Faculty agreed to work together to identify gaps in implementation science and to develop and deliver new presentations at the training institute to be published subsequently for the broader field. Moreover, our trainees would be expected to contribute to implementation science’s knowledge base. Third, we perceived implementation of evidence-based mental healthcare as uniquely complex in some ways, given its array of specialty and non-specialty platforms and treatment types (acute care, pharmacology, psychosocial). Such complexity would demand a longer than typical training period; thus, we planned to provide each cohort with two years of annual immersion experiences, enriched by *in vivo* training through site visits to state-of-the-art implementation research projects specifically selected for fellows’ emerging research programs.

Several other assumptions shaped the training plan. Because implementation science is inherently multidisciplinary, IRI faculty, fellows, and curriculum content would necessarily draw on several disciplines. Similarly, implementation is a multi-level phenomenon, with distinct but interacting and nested processes operating at the individual-, team-, organizational-, and policy-context levels [8,9]; this should shape the curriculum and selection of faculty. We felt that the training should have strong national leadership, given the existence of only pockets of expertise in implementation science around the country. No university-based departments were leading the field or had a sizable ‘critical mass’ of implementation researchers among their faculty and students. Accordingly, we expected that most trainees could draw on mentors in their home institutions for grant-writing expertise, but probably not expertise in implementation science. Finally, the IRI is predicated on a pedagogical philosophy that values interpersonal activity and interaction as a key to the science-building process [10]. Therefore, our training would provide opportunities for interpersonal networking among not only faculty and trainees but also other types of mentors and stakeholders in real-world clinical and service settings.

These assumptions led us to design the IRI to address two overarching goals: first, to strengthen human capital for the field of implementation science through the training of a new generation of implementation researchers, and second, to advance intellectual capital for the still-developing field—through stimulating the production of scholarly products such as papers, books, and curriculum models. While the first goal is typical of early investigator training programs, the second goal was considered necessary because of the emerging science status of the implementation research field. Moreover, the IRI was purposively shaped to differ from many other training grants in that the purpose is not to teach grant writing to very junior scholars, but rather, to help both promising and proven scholars make a successful transition to implementation research.

Guided by these assumptions and the assessment of need, the authors of this paper shaped an R25 proposal, first submitted to the NIMH in 2007 and eventually funded in 2009. The IRI was established with core funding from the NIMH R25 grant and supplementary funding from the Department of Veterans Affairs (VA). This interdisciplinary training program provides didactic training, faculty mentoring (both local and distance), support and guidance for pilot research and grant writing, and additional experiential learning—all focused on helping participants develop implementation research projects for competitive external funding. Part of the experiential learning involves observation of agency- and research- implementation efforts in mental health settings. Fellows are PhD and/or MD investigators from a variety of health-related fields, with demonstrated experience and enthusiasm in the study of mental healthcare, who wish to conduct ground-breaking research in the area of implementation science. Next, we describe the IRI components, outcomes to date, and participant experiences with the Institute.

## Methods

Data shown below was approved by the Washington University Human Research Protection Office (IRB ID #: 201204044). Informed consent was exempt for this study.

### Guiding definitions

The Institute is guided by definitions used by the NIH. Specifically, we understand implementation as ‘the use of strategies to adopt and integrate evidence-based health interventions and change practice patterns within specific settings’ [11] and implementation research as scientific investigations that support movement of evidence-based, effective healthcare from the clinical knowledge base into routine use [12]. While the IRI focus is on implementation research, content addresses dissemination research as well, defined by the NIH as ‘the study about how, when, by whom and under what circumstances research evidence spreads throughout the agencies’ [13].

### IRI participants

#### Leadership

The Institute is led by a Director, Associate Director, and a Coordinator. Enola Proctor, PI of the NIMH R25 grant, is IRI Director. The Associate Director is John Landsverk, also Director of the Child and Adolescent Services Research Center in San Diego. Drs. Proctor and Landsverk bring to their leadership roles several decades of experience directing research centers, implementation research studies, providing academic administration, and leading formal training programs (including NIH T32 programs). A PhD level Coordinator, Ana Baumann, is based at Washington University in St. Louis, as is Dr. Proctor. Together they lead Institute program administration, including fellow recruitment; planning and organizing training events, and evaluation of the program.

### Core faculty

In addition to Proctor and Landsverk, four nationally renowned implementation researchers were recruited to serve as members of the IRI Core Faculty for the five years of the funding period: Gregory Aarons, University of California, San Diego (Psychiatry); Ross Brownson, Washington University in St. Louis (Public Health); Charles Glisson, University of Tennessee (Social Work); and Brian Mittman, Department of Veterans Affairs (Management Theory). Each has an established, externally funded research program relevant to the implementation of evidence-based practice (EBP) and a strong record of training postdoctoral fellows and junior faculty for independent research careers.

Members of the Core Faculty work as a group to chart the long-range direction of the IRI, assess adequacy and gaps in training aims, and identify opportunities for collaboration and partnership. They constitute the fellow selection committee and each serves as primary mentor for specific fellows. The Core Faculty accomplish these functions through virtual and in-person meetings throughout the year. Each leads one or more sessions at the annual Institute. Program staff from the NIMH and VA are invited to each Institute, participating as Expert Faculty. The NIMH Associate Director for Dissemination and Implementation Research, Dr. David Chambers, has made presentations each year. Additionally, he has contributed actively between Institutes to requests from Core Faculty to help identify potential expert faculty and sites for learning visits.

To inform curriculum revisions, the Core Faculty hold periodic telephone and in-person meetings, including one meeting during each Institute focused specifically on assessing the curriculum and identifying new areas of emphasis in future years. Curriculum reassessment is also informed by annual evaluations performed by both faculty and fellows.

### Expert faculty

For each annual Institute, an additional eight to ten individuals are invited to serve as Expert Faculty, chosen by IRI Directors and Core Faculty for their ability to bring expertise in targeted curricular areas at the Institute. The composition of Expert Faculty changes from year to year, guided by the Core Faculty’s on-going assessments of curricular needs and progress in the field. Members of the Expert Faculty illustrate their ongoing mental health implementation research in a range of private, VA, and other public community and acute care settings. In the first three years, Expert Faculty have represented the disciplines of clinical psychology, organizational psychology, sociology, communication theory, medical anthropology, psychiatry, epidemiology, medicine, social work, public health, and electrical engineering (see Table 1 for details). Expert Faculty typically spend two to three days at the Institute, networking and advising fellows in addition to lecturing on specific topics.

**Table 1:** Disciplines from core and expert faculty, years 2010, 2011 and 2012

**Discipline**	**Number of people**
Medicine	6
Psychology^a^	6
Social Work	4
Psychiatry	3
Sociology	2
Medical Anthropology	2
Public Health	2
Epidemiology^b^	3
Organizational Behavior and Management	1
Communication Therapy	1
Electrical Engineering, Mathematics	1
Administration	1

Note: ^*a*^five faculty from Psychology, and one faculty from Industrial/Organizational field; ^*b*^one faculty from Epidemiology, one faculty from Epidemiology and Public Health, and one from Nutrition Intervention and Policy; Epidemiology.

### Fellows

Ten fellows are selected each year, eight supported by the NIMH funding and two by VA funding. Information about the IRI and application process is widely disseminated through relevant email distribution lists, listservs, word-of-mouth, and conference announcements; the application period is open for four months, typically from the end of October to February. The application requires a letter describing the applicant’s interest in implementation research in the context of their career goals and long range research agenda, an initial concept paper in the area of mental health implementation science that the applicant expects to advance through the IRI toward submission to a federal funding agency, a description of resources and supports in his or her home institution for grant-writing fundamentals and for authoring scholarly publications, and a letter from a local mentor in their home institution. Selection criteria include: prior or concurrent experience relevant to IR such as intervention development and/or testing, mental health services research, or study of organizational factors in mental health service delivery; experience writing an NIH, VA, or other federal grant; a strong local mentor in the applicant’s home institution who is supportive of the fellow’s grant writing and scholarly publication (the local mentor need not be an expert in IR, but must have a strong record of NIH or VA funding); and access to a clinic/service setting willing to serve as a pilot site for the fellow’s implementation research. Applications are rated using the NIH grant review criteria where Core Faculty review applicant’s current research for evidence of a ‘path’ toward dissemination and implementation research, prior accomplishments (training agenda vitae, previous funding, letters of recommendation/support), and potential for a productive implementation research career. Preference is given to applicants who have NIH or VA grant-writing experience, have secured funding (*e.g.*, F32, K, R03, R34, R21, R01 or similar mechanisms in the VA) in fields related to implementation research, and demonstrate potential to succeed in the Institute. Core Faculty independently score each application and final selections are made during a conference call, wherein they discuss applications, finalize selections, and make preliminary assignments of primary mentors. Applicants and selected fellows are notified within a few days of the decision, and each selected fellow is asked for a commitment to attend two consecutive Institutes. IRI completion requires full participation in the two-year program.

Selected fellows comprise a diverse group of participants from various disciplines (*i.e.*, psychology, social work, pediatrics, psychiatry, anthropology; epidemiology; see Table 2 for more details), institutions, and geography to build a national network of implementation researchers. All IRI fellows have entered the program with at least one externally-funded grant, primarily to eliminate the need to address basic grant writing and enable it to focus on implementation science. While all fellows enter the IRI at early stages of implementation science knowledge, they vary in terms of career stage and prior accomplishments. Fellows range from one year out of their terminal degree programs (PhD, post-doc, or M.D.) to Full Professor. Several entered with more limited publication records (minimum number of publications for an entering fellow to date is 13), but more experienced fellows had more than 50 publications. Fellows similarly vary in prior research grant experience. Many enter with one funded grant (and for one fellow, this was an F31 dissertation grant), while several have received R03’s, R34’s, and career development awards. However, none of these grants were in implementation science. This marked variation in background and experience poses challenges for the development and delivery of the training program, as detailed below.

**Table 2:** Disciplines from 2010, 2011, and 2012 IRI fellows

**Discipline**	**Number of people**
Psychology^a^	19
Social work	4
Medicine	3
Psychiatry	2
Epidemiology	1
Anthropology	2

Note: ^*a*^ fifteen fellows are from Clinical Psychology, one from Social Psychology, one from Counseling Psychology, one from Health Psychology, one from Applied Experimental Psychology.

### IRI components

#### Annual institute

The IRI’s key training component is an annual five-day Institute each summer at Washington University in St. Louis. The Faculty considered core content areas required in implementation science as they wrote the R25 grant proposal and published a guiding heuristic framework for implementation research in mental health [8]. This framework served as the model for our identification of core areas, which have remained constant over the first three Institutes. The core areas include: theoretical bases of implementation science, and ability to select, critique, and use an established conceptual model or framework to guide a research study; the distinction between, and the evidence for, two practice-change technologies: clinical/behavior change interventions, and implementation strategies; contextual factors influencing implementation, such as organizational context and leadership, and the ability to understand whether a given study will observe, control, or manipulate those factors; the multi-level nature of practice or service system change, and ability to select appropriate research methodologies, including randomized control designs and alternatives to group randomization, measurement of implementation strategies and outcomes, multilevel modeling, mixed methods including community based participatory approaches, and alternative sources of data for implementation research; understanding key partners in implementation research and skills required to engage them in programs of implementation research; and mounting IR programs of research; and understanding of and ability to address the specific ethical issues in IR and capacity for responsible conduct of science.

Specific topics within these key areas are adjusted from year to year according to advances in the field, the unique interests and experience level within each IRI cohort, and the presence of new and returning fellows each summer after the first year. For example, in year one, Expert Faculty member James Dearing gave an overview of Rogers’ Diffusion of Innovation Theory. The ensuing discussion raised the challenge of understanding, critiquing, and selecting a particular theory or conceptual model given the tendency at that time for implementation researchers to develop unique models for each grant application. Accordingly, for the year two Institute, David Chambers and Ross Brownson developed and presented a session on frameworks used in NIH funded D&I research. From this session, they collaborated to write and publish a paper overviewing and comparing 62 models and theories—a paper that became required reading thereafter [14].

Given the multidisciplinary and rapidly evolving nature of implementation science, the Core Faculty spends nearly a full year planning each year’s curriculum. The Core Faculty keep abreast of developments in implementation science for mental health, consult with NIMH program officers, and nominate and invite new Expert Faculty based on their own learning and experiences throughout the year. To accelerate fellows’ appreciation of the realities of conducting implementation research, the curriculum draws heavily on current examples of funded research in the field. Each year’s curriculum is also shaped by fellows’ needs, interests, and characteristics. The format and combinations for didactic and experiential teaching methods is informed by evaluations of the previous Institutes. Each Institute employs a variety of learning formats, including didactic presentations by faculty, presentations by fellows, peer critique and feedback, informal networking, small group interactions, and brief ‘consultation with the faculty’ sessions arranged through a sign-up process. The week-long Institute in St. Louis is an ‘immersion’ experience providing opportunity for strategic networking. For example, each Institute provides an afternoon for fellows to consult with Core and Expert Faculty on a sign-up basis, and Faculty are available during evening hours for further consultation on the grant proposals fellows develop during the Institute.

Since the second year, each Institute comprises a cohort of returning ‘second year’ fellows along with a new cohort of ‘first year’ fellows. We assume that first year fellows, albeit with research experience and some grant writing success, may be relative ‘novices’ in IR, while second year fellows come to their second Institute at a more advanced level. Accordingly, the curriculum is structured to provide optimal redundancy but minimal repetition of basics for second year fellows [15,16]. For all topics that are repeated in the second year (such as research designs for implementation studies), the particular focus, examples, readings, exercises, and expert faculty differ significantly to convey emerging issues and recent advances in implementation science. This approach increases depth and breadth of perspective across the two years that each fellow experiences. Second year fellows are engaged in some teaching [17], and actively report on their interim learning. Over the five intensive days, each fellow is expected to advance a concept paper (first year fellows) or grant proposal (second year fellows), developing aims and research questions for mental health implementation science, shaping conceptual models, and planning details for the study methodology. During the Institute, second year fellows receive extensive and critical reviews of their proposal by reviewers experienced in the NIH review process. These reviews serve to normalize the process of critical feedback, sharpen ability to read critically, facilitate progress in writing proposals, enable students to send draft proposals to NIMH staff for feedback, and ultimately support success with external funding. By the week’s end, all fellows are expected to have developed an action plan for advancing their IR grant proposal. Fellows’ presentations and revised plans for grant proposal development serve as the reflection, within the Institute, of their understanding of core content areas and their preparedness for implementation research.

### Pre-institute preparation

Approximately three weeks before the Institute, each fellow and faculty receives an electronic course packet containing IRI objectives, a biographical sketch of each participant, readings that correspond to each session, and the training agenda. Core faculty receive first year fellows’ IR project concept paper; and second year fellows and faculty receive the fellows’ grant proposals for review. Second year fellows also provide their report of learning site visits with Core Faculty (see below). The content of these reports are embedded in the curriculum of the Institute as current examples of IR studies. The packet launches distance learning and reduces the burden for both faculty and trainees during the Institute.

### Mentoring

Fellows are matched with an Institute mentor (one of the six Core faculty members) with whom they meet by phone- or video-conference call monthly to bi-monthly during the two year training. Mentoring focuses on the progress of the pilot study, the development of the fellow’s IR research proposal, and feedback from NIH program staff around the developing grant proposal. To facilitate continued application of content from the Institute, all fellows and faculty have access to the videos of the lectures from all previous IRI years via a secure website.

### IR site visits

Good research training requires the opportunity to learn from projects that are scientifically innovative, important, and challenging [18,19]. We view active, still-in-the-field projects that have passed stringent peer review as an excellent source of such learning. Therefore we provide each first year fellow with travel funds to support a two to three day visit to an active implementation research site supported by highly competitive federal funding. Working closely with NIH and VA program officers and informed by NIH Reporter searches, Proctor, Landsverk and Baumann identify sites of state-of-the-art IR that reflect good science and partnership models. See Table 3 for examples of learning sites that fellows have visited.

**Table 3:** IRI learning sites

**PI**	**Title**	**Funding source**	**Location (Organization State)**
Sonya Leathers	Dissemination of effective MH services in foster care	NIMH; R01	Chicago, IL
Philip Kendall	Disseminating evidence-based practice to the schools: CBT for child anxiety	NIMH; R01	Philadelphia, PA
Madhukar Trivedi	Using information technology to provide measurement based care for chronic illness	AHRQ; R18	Dallas, TX
Joann Kirchner	Blended facilitation to enhance PCMH program implementation	VA	Little Rock, AR
John Weisz	Youth mental health network	The John D. and Catherine T. MacArthur Foundation	Honolulu, Hawaii, Boston, Mass.
Jeffrey Epstein	Evaluation of an intervention for improving community-based pediatric ADHD care	NIMH; R01	Cincinnati, OH
Geoffrey Curran	Training SUD treatment counselors CBT for depression	VA	Little Rock, AR
Gregory Aarons	Organizational issues in implementing EBP in child welfare and mental health	NIMH	California, San Diego
Gregory Aarons	Mixed-methods study of EBP sustainment in Statewide service system	NIMH; R01	California, San Diego
Mark Chaffin	SafeCare home-based service models	NIMH; R01	Oklahoma City, OK
Patricia Chamberlain	Experiment in implementing multidimensional treatment foster care	NIMH/NIDA R01	California, Ohio
John Fortney	Partnership for implementation of evidence-based practices in rural primary care	NIMH; R01	Little Rock, AR
Kimberly Hoagwood	Implementation of feedback system to improve EBTs for children in mental health	AHRQ; R18	New York
Richard Owen	Monitoring and Management of Metabolic effects of Antipsychotics	VA	Little Rock, AR
Alex Young	Implementing effective, collaborative care for Schizophrenia	VA	Los Angeles

Prior to their visits, we provide webinar training to all fellows to optimize their ability to learn from these visits. Each fellow completes two assignments from these visits: a site visit report on topics deriving from the training agenda, including key concepts (is the project conceived as an implementation study, an adaptation of an EBP, dissemination, or test of practice effectiveness?), the study’s conceptual model (explicit or implicit), the EBP(s) being implemented, the implementation processes or strategies in place, implementation barriers, the implementation agency’s characteristics, clinical and implementation outcomes measured [8], the type and elements of the design employed [20], and the stakeholders and partnerships exemplified in the project; and a scientific biography of the IR study’s PI, including educational history and training, transition points evident in his/her research career, funding history and key publications, and team composition. When they return for their second year Institute, these ‘senior’ fellows share with other fellows the conceptual and methodological developments apparent in the implementation studies they have visited. Second year fellows’ feedback guide revisions and updates to the IR site visit assignments. The site visits also enable Core Faculty to identify an emerging network of IR investigators to include in the IRI as possible Expert Faculty.

### Pilot research in real-world implementation

Each fellow receives funding to conduct pilot work in a clinic/service setting that is implementing EBPs and is willing to serve as a pilot study site. IRI Faculty help negotiate fellows’ agency experiences. The formal curriculum and faculty mentoring guide trainees through IRB issues and processes associated with IR. This component of the IRI initiates fellows to the partnerships with real world sites required of all implementation researchers, and the relationships that are fostered are expected to develop into longer-term, grant-related partnerships.

### Face-to-face meeting for networking at an IR conference

First and second year fellows receive support to attend an implementation science conference, such as the NIH Conference on the Science of Dissemination and Implementation in Health Implementation or the Seattle Implementation Research Conference (SIRC). All Core Faculty are encouraged to attend the conference, which provides a platform for formal presentations of fellows and faculty’s work, as well as informal gatherings. Core Faculty and fellows attend an informal networking dinner, where they network and recognize graduating ‘senior’ fellows for their accomplishments and contributions.

**Figure 1:**
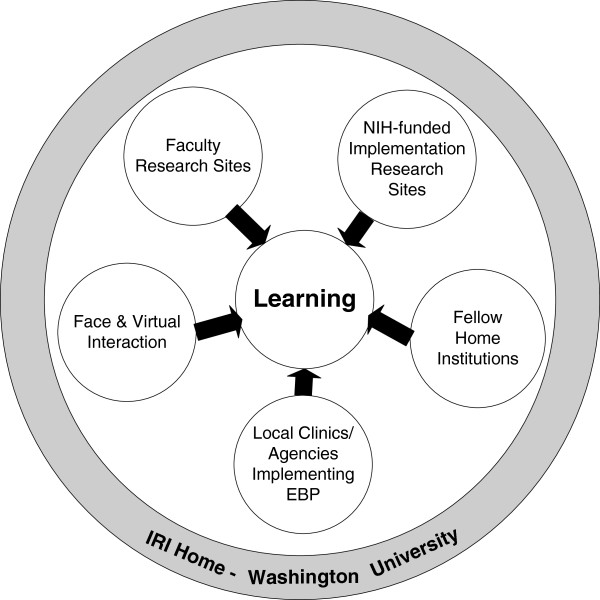
IRI training environments.

In summary, conceptualizing implementation science as multi- and trans- disciplinary, the goal of the IRI is to ensure that each fellow benefits from a confluence of six training environments during their two years of training, as shown on Figure 1: the IRI host site, the Washington University Center for Mental Health Services Research (CMHSR)—the ‘intellectual and training hub’ and the coordinating center; the fellow’s home environment, which will commit to mentoring and supporting grant development; national sites of NIH-funded implementation research hosting fellows’ learning site visits, including but not limited to the research settings of some IRI Faculty, generating new advances in implementation science; local clinics/agencies settings as pilot sites for fellows; and interim face-to-face and virtual collaborative interactions.

### IRI evaluations

IRI processes and outcomes are evaluated for fellows, mentors, and the program. After each Institute, all fellows and faculty are asked to evaluate the training and provide feedback on the logistics of the institute (*e.g.*, organization of the schedule, clarity of instructions), on the formal training (*i.e.*, on each of the sessions), on the informal training (*e.g.*, networking, caliber of the fellows), and on mentoring (*e.g.*, have fellows received feedback on their projects? Have faculty provided feedback on fellows’ projects?).

## Results

Our evaluations show that fellows and faculty have been very satisfied with the program and would recommend IRI to colleagues. Fellows and faculty have rated the caliber of their counterparts as excellent on all three initial years (See Table 4 for more details). Participant feedback regarding the program has shaped the next year’s curriculum. For example, because fellows and faculty requested more time to network and ‘digest’ the contents learned for their own projects, we reduced the number of didactic classes per day, and increased formal and informal opportunities to network and discuss research projects with faculty and peers.

**Table 4:** Average rating from 2010, 2011, and 2012 IRI evaluations by fellows

	**Average**	**SD**	**Range**
**Logistics**			
Timeliness of acceptance decisions	4.67	0.47	3–5
Adequacy of information I received prior to the IRI to prepare me for the IRI	4.49	0.65	3–5
Organization of the IRI schedule	4.29	0.65	3–5
**Informal Training**			
Professional caliber of other fellows	4.96	0.20	4–5
Collaboration with fellows	4.67	0.52	4–5
Interactions with non-core faculty	4.65	0.60	3–5
**Mentoring**			
Likelihood of pursuing further professional relationships with faculty and fellows	4.88	0.39	3–5
Interactions with core faculty	4.76	0.52	3–5
Helpfulness of feedback from faculty on pilot and NIH proposal	4.66	0.60	3–5
Helpfulness of feedback from faculty on my ideas	4.63	0.60	3–5
Helpfulness of peer support from other fellows on ideas	4.47	0.68	3–5
Assistance received with study design	4.30	0.72	3–5
**Overall**			
Likelihood of recommending IRI to colleagues	4.92	0.35	3–5
Satisfaction with IRI program	4.84	0.42	4–5

Note: 1 = very poor; 2 = poor; 3 = fair; 4 = good; 5 = very good.

The overall goals of the IRI are to concurrently advance the science of implementation, especially in the area of mental health, and develop the supply and capacity of implementation researchers. Accordingly, we posit outcomes at three levels: participating fellows, participating faculty, and for the fields of mental health and implementation research. This paper overviews outcomes to date in each category. At the end of year five, we will prepare a more detailed analysis and findings regarding these three levels of outcomes for a subsequent paper for publication.

### Outcomes for participating fellows

Over its first three years, the IRI has recruited three cohorts for a total of 31 fellows (in the first year, IRI had 11 slots, followed by 10 slots for years two and three). These fellows have been selected from a total pool of 86 applicants from a wide range of academic settings and disciplines as shown in Table 2. Fellows from the first three cohorts (n = 31) completing their two-year training periods (2009–2011, 2010–2012, and 2012–2013) have submitted 74 proposals for scientific projects and received 52 (70%) funded awards. The funding mechanisms varied: 21% were NIH R01 grants, 21% VA projects, 11% NIH R34 grants, 8% NIH K grants, and the remaining 34% are grants from other mechanisms, such as CDC, AHRQ, and Fogarty (Please see Table 5). The new research supported by the IRI training will generate new findings in the emerging field of implementation science with promise to accelerate the availability and quality of evidence-based mental healthcare.

**Table 5:** Outcomes of 2010, 2011, and 2012 cohort of IRI fellows

**Outcomes**	**Numbers**	**Additional information**
Grants	Number of grants submitted: 74	Percentage of type of grant awarded:
		21% NIH R01
	Number of grants awarded: 52 (70%)	21% VA projects
		11% NIH R34
		8% NIH K
		39% other sources or mechanisms (*e.g.*, NIMH R21, SAMSHA, AHRQ, CDC, Forgaty, Melinda and Bill Gates Foundation)
Publications	Total number of publications: 208	
Other accomplishments	63 presentations at conferences and other accomplishments	

Within the first year (for the first two cohorts of fellows) and six months (third cohort) after completing the IRI, the three cohorts submitted and/or published a total of 208 publications (mean of 7.64 publications per fellow) related to the field of implementation science. They also made national presentations in the VA QUERI Cyber Seminar series and at numerous conferences, and delivered seminars and lectures in their local institutions. These contributions reflect IRI fellows’ preparedness to address the conceptual, methodological, and practical challenges of studying the implementation of evidence-based mental health interventions. While the publication and grant award rates for IRI fellows are clearly impressive, we urge caution in the attribution of accomplishments to the IRI. An explicit aim for year five of the IRI award is to conduct detailed evaluations by cohort in an effort to identify fellow and training factors associated with various types and trajectories of outcomes. Those analyses will cover a longer period of time, which will capture fellows’ outcomes beyond their first year. We fully expect their contributions to grow over time.

### Outcomes for participating faculty

In its first three years, the IRI has recruited 27 Expert Faculty from a wide range of disciplines (see Table 1) to join the six Core Faculty in providing Institute training. Each faculty member leads one or more sessions in the week-long Institute and works to establish relationships with each other and with fellows. For example, although we ask Expert Faculty members to spend only two days at the Institute (whereas Core Faculty members attend all five days), each year several experts have extended their stay and actively participated in discussion, networking, and mentoring of IRI fellows. Faculty and fellows have collaborated in manuscripts, and Core Faculty are primary sponsors, mentors or consultants on several of the fellows’ grants. Noting the emergence of collaborations, we launched an effort, still ongoing, to formally measure ties through an ongoing social network study.

IRI Core faculty members have authored papers advancing specific conceptual, substantive, or methodological issues in implementation research. For example, Brownson and Proctor together with Expert Faculty member Colditz edited a first-of-its kind book on dissemination and implementation research in health [7], a book for which eight other IRI faculty members contributed chapters. All Core Faculty and the IRI NIH program officer, David Chambers, also published a conceptual model for the study of implementation processes [8]. Other papers published by core faculty include a critical analysis of the concept of implementation outcomes [21], a compilation of strategies for implementation research [22] a review of the dissemination and implementation theories and frameworks [14], a review of research methods relevant to implementation science [20], a paper on hybrid designs for implementation research [23], and an implementation conceptual framework specific to public sector mental health and allied health services [24]. Thus, consistent with the original goals, IRI faculty are advancing both the intellectual capital and the workforce capacity for the field of D&I research.

### Outcomes for the field

The IRI has stimulated and informed other training in dissemination and implementation science. The IRI curriculum is publicly available at (http://cmhsr.wustl.edu/Training/IRI/Pages/ImplementationResearchTraining.aspx). In 2010, a planning group developing NIH-wide training for dissemination and implementation research requested detailed information about the IRI. The IRI application process and Institute curriculum provided a model for the Training Institute on Dissemination and Implementation Research in Health (TIDIRH), launched in 2011 with VA collaboration and support. The TIDIRH is an annual, week-long training institute for researchers interesting in learning about dissemination and implementation research for a variety of health conditions and fields. TIDIRH is designed to encourage researchers new to D&I research to develop applications to the trans-NIH Funding opportunity announcements on D&I Research and related VA funding opportunities [25]. Each year, approximately 30 to 45 fellows participate in TIDIRH, hosted by an academic organization with strength in D&I research, led by a Core Faculty of National D&I research experts, and including guest faculty around key topics of interest [25]. The TIDIRH program is targeted to researchers at earlier stages of development than IRI. In fact, investigators that have received R01 or equivalent in implementation science are not encouraged to apply. Two individuals have first completed TIDIRH, then participated in IRI. IRI faculty serve as core (RCB, EKP, BSM) and visiting faculty members (GAA) for the TIDIRH program.

IRI Core Faculty contribute to other training initiatives in dissemination and implementation research: Mittman provides leadership for VA Enhancing Implementation Science (EIS) program; Aarons has helped plan and deliver presentations for the NIH/OBSSR workshop on the use of mixed methods in implementation research, and Proctor and Mittman have led sessions on training at the NIH Meeting to Advance the Science of Dissemination and Implementation. Finally, other impacts should be noted, such as faculty and fellows’ contributions to their colleagues’ work (*i.e.*, implementation science consultation/expertise), presence and leadership of Core Faculty in both IRI and TIDIRH, along with local teaching and mentoring. Fellows have mentored their peers, and faculty experiences in IRI have shaped and strengthened their own local teaching to non-IRI trainees and junior colleagues in countless ways.

### Challenges and limitations

Designing and consistently delivering high quality D&I training on a national level poses many challenges. A primary challenge is that of training for a still evolving and rapidly developing field. To meet this challenge, Core Faculty have been vigilant to monitor developments in the field, including key publications, newly funded research studies, and opportunities for networking and research funding. The Core Faculty also work to identify gaps in the literature, and address them by authoring papers themselves and delivering cutting edge content at each Institute. For example, Aarons and Glisson have presented on still-in-the field research on leadership for implementation and a multi-site trial of the ARC model, respectively.

A second challenge is providing mentoring and support at a distance, since except for the two summer weeks at Washington University IRI fellows are based at their home institutions. Many local mentors are not conducting and may be unfamiliar with dissemination and implementation research. We address this challenge as follows. Core Faculty mentors meet regularly with their mentees via phone, skype and email communication, and at national and local meetings and conferences. Use of these communication channels has facilitated ongoing mentoring even while mentors or fellows are out of the United States. Often, the fellow and the local mentor participate together in the mentoring meetings.

A third challenge is meeting the high demand for training. Each year, the pool of applicants has been extremely strong in terms of experience in IR and successful applications for federal funding—stronger than anticipated when the IRI was first designed. We have responded to this demand by working actively with potential applicants, deterring many individuals who are keenly interested by not yet prepared for the very competitive pool. We also refer many potential applicants to TIDIRH, given the different selection process and scope of the training programs. The resultant high caliber of selected fellows contributes to the collegiality of the IRI, because some fellows are mid- to senior level researchers themselves.

A fourth challenge, more pronounced in the first year, was securing the agreement of IR learning sites to host fellows. The Core Faculty found it challenging to accurately communicate the purpose and nature of the learning visits, and some potential hosts declined participation because of the time burden and the worry that their current research projects and processes would be exposed to ‘outside’ visitors. This challenge has been addressed through refinements in communication, as well as the nearly uniformly positive experiences of fellows and host researchers alike.

Another challenge, indirect to IRI but very real to its long-term success, is preparing researchers new to implementation research within the currently challenging funding environment. Low pay lines make it difficult for scholars starting in a new field to get their grants funded. The pilot funds provided to fellows gives some, albeit very limited, support for preliminary IR studies.

A final challenge is the heavy administrative burden of administering the program, given the low indirect rate and stringent caps on administrative and personnel expenses allowed for R25 mechanisms. The major aspects of IRI are the Institute, the site visits and the travel to a dissemination conference. Something as basic as ensuring correct reimbursement of these trips entails heavy administrative work due to the large number of fellows and faculty and the frequency of travel. Large administrative time is also spent preparing applications for review and ensuring that all Core Faculty have the required materials for review.

Despite the challenges, the IRI provides opportunities for researchers from a variety of disciplines to gain new perspectives on IR and the rewards brought by multidisciplinary learning experiences. Senior researchers with considerable experience in IR have expressed excitement about getting a full exposure to the range of IR expertise and topics brought by Expert Faculty, even some who are internationally based. There has been a deepening of collegiality and shared excitement in grappling with the gaps in the field and growing approaches to those gaps. Each cohort of fellows has developed multiple joint publications and proposals that would not have happened without the intensive learning and dialogue possible in their IRI cohort and even cross-cohort experience.

### Conclusions and discussion

Bold and innovative efforts are required to develop skilled implementation researchers. To accelerate the development of both human and intellectual capital, the authors of this paper conceived and launched the IRI. As a learning collaborative, the IRI has coalesced senior health and mental health services researchers, treatment developers, expert methodologists, and junior researchers around their shared commitment to advancing their own knowledge of implementation research methods (human capital) and the field itself (intellectual capital). The IRI has now trained 31 new implementation researchers. Fellows have received two years of mentoring with the goal of developing new implementation science research projects. Fellows are engaged in in-depth examinations of conceptual and methodological issues, are exposed to real world implementation efforts and state-of-the-art implementation research, and receive financial support for their own pilot studies. Based the number of implementation research grant proposals and papers produced by fellows to date, the IRI is proving successful in preparing new researchers who can inform the process of making evidence-based mental healthcare more available through real-world settings of care and who are advancing the field of implementation science.

Since the launch of the IRI, several other training programs and infrastructure development initiatives have been launched. Implementation research and training has been pioneered by the VA as part of the Quality Enhancement Research Initiative (QUERI) [26]. The VA Center for Implementation Practice and Research Support (CIPRS), a Los Angeles-based resource center established in 2008, offers annual training in D&I science through its Enhancing Implementation Science (EIS) program, individualized consultation through a helpline and Implementation Research Clinic, an Implementation Research Cyber Seminar series, individual seminars and workshops, and various tools and resources to foster development and growth of implementation research capacity. Two of the NIH supported Clinical and Translational Science Award (CTSA) programs offer specialized training and infrastructure support for D&I science: Washington University’s CTSA program has a Dissemination and Implementation Research Core (DIRC), and the University of California San Francisco’s CTSA offers an Implementation and Dissemination Science (IDS) Certificate Program, a Master’s Program in Clinical Research, and a Ph.D. in Epidemiology and Translational Science whose conceptual framework, training domains, courses, and competencies are described in an article by Gonzales *et al*. [27]. The Canadian Knowledge Translation (KT) training initiative began as a KT Summer Institute in 2008, and its conceptualization, core competencies, and ‘lessons learned’ are described in several recent publications [28-31]. Straus *et al*.[31] note their inability to find national training strategies which their program could model, reflecting the need for a literature on implementation science training—a literature to which the present paper contributes, as does the Stamatakis *et al*. [32] paper on insights from recent implementation science trainees, one of whom is an IRI ‘alum’.

The final year of the current funding cycle will support the Core Faculty in assessing various curriculum models that evolve from the IRI experience, identifying new topics and lessons learned that will be developed to inform and support training experiences for trainees earlier in the pipeline, such as doctoral and postdoctoral students, and potentially for earlier levels where more specific and limited core competencies are appropriate. It also will support collection of outcomes over a longer observation period, as well as more nuanced analyses of outcomes and identification of factors associated with varying levels of fellows’ contributions to implementation science.

## Abbreviations

IRI: Implementation Research Institute; NIMH: National Institute of Mental Health; VA: Department of Veterans Affairs; NIH: National Institute of Health; OBSSR: NIH Office of Behavioral and Social Sciences Research; EBP: Evidence based practice; CMHSR: Washington University Center for Mental Health Services Research; CDC: Centers for Disease Control and Prevention; AHRQ: Agency for Healthcare Research & Quality; TIDIRH: Training Institute on Dissemination and Implementation Research in Health; QUERI: Quality enhancement research initiative; CIPRS: The VA Center for implementation practice and research support; EIS: Enhancing implementation science; CTSA: Clinical and translational science award; DIRC: Washington University’s CTSA Dissemination and Implementation Research Core; IDS: University of California San Francisco’s CTSA Implementation and Dissemination Science; KT: Canadian knowledge translation.

## Competing interests

The authors declare that they have no competing interests.

## Authors’ contributions

EKP and AB outlined the paper and developed initial drafts. All other authors contributed directly to writing the article. All authors read and approved the final manuscript.

## Authors’ information

EKP directs the Center for Mental Health Services Research at Washington University in St. Louis (NIMH P30 MH085979), the Dissemination and Implementation Research Core (DIRC) of the Washington University Institute of Clinical and Translational Sciences (NCRR *UL1RR024992*), and the Implementation Research Institute (NIMH R25 MH080916).
